# Does TomoDirect 3DCRT represent a suitable option for post-operative whole breast irradiation? A hypothesis-generating pilot study

**DOI:** 10.1186/1748-717X-7-211

**Published:** 2012-12-14

**Authors:** Valeria Casanova Borca, Pierfrancesco Franco, Paola Catuzzo, Fernanda Migliaccio, Flora Zenone, Stefania Aimonetto, Andrea Peruzzo, Massimo Pasquino, Giuliana Russo, Maria Rosa La Porta, Domenico Cante, Piera Sciacero, Giuseppe Girelli, Umberto Ricardi, Santi Tofani

**Affiliations:** 1 Departments of Medical Physics, Ospedale Regionale ‘U. Parini’, AUSL Valle d’Aosta, Aosta, Italy; 2 Departments of Medical Physics, Azienda Sanitaria ASL TO 4, Ivrea, Italy; 3 Department of Radiation Oncology, Ospedale Regionale ‘U. Parini’, AUSL Valle d’Aosta, Aosta, Italy; 4 Departments of Radiotherapy, Azienda Sanitaria ASL TO 4, Ivrea, Italy; 5 Department of Medical and Surgical Sciences, Radiation Oncology Unit, University of Torino, Ospedale S. Giovanni Battista, Turin, Italy

## Abstract

**Background:**

This study investigates the use of TomoDirect^TM^ 3DCRT for whole breast adjuvant radiotherapy (AWBRT) that represents a very attractive treatment opportunity, mainly for radiotherapy departments without conventional Linacs and only equipped with helical tomotherapy units.

**Methods:**

Plans were created for 17 breast cancer patients using TomoDirect in 3DCRT and IMRT modality and field-in-field 3DCRT planning (FIF) and compared in terms of PTV coverage, overdosage, homogeneity, conformality and dose to OARs. The possibility to define patient-class solutions for TD-3DCRT employment was investigated, correlating OARs dose constraints to patient specific anatomic parameters.

**Results:**

TD-3DCRT showed PTV coverage and homogeneity significantly higher than TD-IMRT and FIF. PTV conformality was significantly better for FIF, while no differences were found between TD-3DCRT and TD-IMRT. TD-3DCRT showed mean values of the OARs dosimetric endpoints significantly higher than TD-IMRT; with respect to FIF, TD-3DCRT showed values significantly higher for lung V_20Gy_, mean heart dose and V_25Gy_, while contralateral lung maximum dose and contralateral breast mean dose resulted significantly lower. The Central Lung Distance (CLD) and the maximal Heart Distance (HD) resulted as useful clinical tools to predict the opportunity to employ TD-3DCRT: positive correlations were found between CLD and both V_20Gy_ and mean lung dose and between HD and both V_25Gy_ and the mean heart dose. TD-3DCRT showed a significantly shorter mean beam-on time than TD-IMRT.

**Conclusions:**

The present study showed that TD-3DCRT and TD-IMRT are two feasible and dosimetrically acceptable treatment approach for AWBRT, with an optimal PTV coverage and adequate OARs sparing. Some concerns might be raised in terms of dose to organs at risks if TD-3DCRT is applied to a general population. A correct patients clusterization according to simple quantitative anatomic measures, would help to correctly allocate patients to the appropriate treatment planning strategy in terms of target coverage, but also of normal tissue sparing.

## Background

Adjuvant whole-breast external beam radiation therapy (AWBRT) is an integral part of the current standard multimodality approach for early stage breast cancer [1]. Intensity modulated radiation therapy (IMRT) has been shown to increase intra-target dose homogeneity and to spare organs at risk [2]. Recent studies on breast irradiation [3] have demonstrated that Helical Tomotherapy (HT) provides superior target dose homogeneity and moderate normal tissue sparing compared to conventional treatments. TomoDirect^TM^ (TD) is a fixed beam treatment mode allowing for planning and delivery at static beams, with the couch moving at a constant rate past a fixed binary multileaf collimator for fluence modulation [4]. A pre-clinical version of the planning system named topotherapy has been investigated in the context of AWBRT, showing adequate target coverage and normal tissue high dose reduction with concomitant PTV inhomogeneous dose in one study [5,6]. In addition to the IMRT mode (TD-IMRT), it is possible to plan TD in a ‘3D-CRT mode’ (TD-3DCRT), whereas dose penalties and DVH constraints cannot be specified and a preset optimization algorithm determines fluence maps, resulting in an inherently forward plan still allowing for IMRT. TD-3DCRT might be very attractive, since it provides adequate treatment plans and concomitant significantly lower request in terms of planning, calculation and beam-on time resources than TD-IMRT.

Aim of the present study is to investigate TD-3DCRT in the context of AWBRT in terms of dosimetric outcomes of both target and organs at risk and beam-on time and to compare it to TD-IMRT planning and conventional 3DCRT field-in-field technique (FIF). Additionally we defined appropriate patient-class solutions to employ TD-3DCRT in AWBRT, generating the hypotesis that simple anatomic measures could properly drive patient allocation to an appropriate and cost-effective planning modality.

## Methods

### Patients’ selection, CT simulation and target delineation

The study population comprised 10 left-sided and 7 right-sided breast cancer patients submitted to conservative surgery and AWBRT. Patients underwent 2.5 mm slice thickness CT acquisition in the supine position on a wing-board with both arms abducted alongside the head. Images were acquired from the lower aspect of the mandible to the base of the lungs, with radiopaque wires marking the clinically detectable breast borders. The whole-breast clinical target volume (CTV) encompassed the palpable residual mammary gland with superior and inferior border delimited within the extent of the radiopaque catheters. The subsequent planning target volume (PTV) was generated by adding a 5 mm margin around the CTV but confined to the interior of the patient’s outer contours reduced by 5 mm, also excluding heart and lung when needed. Organs at risks (OARs) contoured were both lungs, contralateral breast and heart, outlined up to the level of the pulmonary trunk superiorly, including the pericardium and excluding the major vessels.

### TD planning

TD-IMRT and TD-3DCRT plans were generated with TomoTherapy Hi-Art version 4.0.4 TPS (Accuray Inc., Sunnyvale, CA). For each plan, the treatment field width, pitch (the TD pitch is defined as the distance of couch travel in centimeters per sinogram projection) and modulation factor need to be selected. Then, the dose distribution for each beamlet that passes through the target is calculated by a convolution/superposition algorithm [7]. Once the beamlet calculation step is completed, the optimization process begins and an iterative least-squares minimization method is used to optimize the objective function. During the final dose computation the optimized sinogram is converted to the delivery sinogram, taking into account for leaf fluence output factors and latency data. A fine calculation grid (256x256 pixels) was used both in the optimization and calculation processes.

The TPS is driven by dose-based objectives, their associated penalties and ROI-based weighting factors. For target volumes, minimum and maximum dose values and their respective penalties are used in addition to a DVH-based prescription point. OARs objectives are described by a maximum dose, a DVH-based constraint and their respective penalties.

Two tangential beams with a jaw width of 2.5 cm were used, that can be considered a suitable compromise between cranio-caudal dose spread (lower than a 5 cm field width) and treatment time (lower than a 1 cm field width). The pitch value was set to the default value that is one tenth of the field width (0.25 cm/projection for the 2.5 cm beam). Beam angles were selected in order to minimize dose to OARs. When you create a beam angle, only those MLC leaves required to treat the target are used.

To help ensure the prescribed dose is delivered to the target taking account for possible target movement for breathing during irradiation, three MLC leaves were opened on the anterior edge of the beams. To calculate the intensity for all leaves required to expand the beam, the average intensity of the last two leaves on the beam edge are used. A ‘fine’ calculation grid was used for both optimization and calculation. In the TD-IMRT mode, a 5 mm ring around the PTV was used to help reduce skin overdosage. Helping structures were created within the body volume outside the PTV where significant hotspots were likely to occur. A complete block was placed on the contralateral breast, if necessary. OARs were used as avoidance structures for the optimization process. A modulation factor of 2.0 was used in all IMRT plans.

The TD-3DCRT mode represents a simpler planning mode, in which the operator does not specify the OAR dose constraints but only the DVH-based prescription for one selected target structure and influences the optimization process only by setting two parameters: the tissue compensation, which refers to the level of beam intensity variation allowed for the plan and may produce more conformal dose distributions but will usually require longer delivery times and the normal tissue homogeneity, which prevents hot spots from forming in normal tissue. The tissue compensation factor can be set as high or low: the high compensation mode uses a modulation factor close to 2, while the low compensation mode uses a modulation factor of approximately 1.

All plans were performed selecting the normal tissue homogeneity option and setting the tissue compensation to high, in order to allow for a more significative comparison with TD-IMRT plans.

The role of tissue compensation and normal tissue homogeneity option has been investigated as well.

In order to keep TD-3DCRT planning mode authors decided not to introduce helping structures, that are intrinsically considered as target volumes by the software and consequently require a careful, accurate and therefore time-consuming countouring; contralateral breast was completely blocked whenever necessary.

### Field-in-field 3DCRT planning

To assess the feasibility of planning whole breast radiation with TD-3DCRT, a comparison with a conventional technique has been performed. A field-in-field 3DCRT planning was used, because it shows a better PTV coverage and uniformity than conventional wedged tangent fields and allows to reduce the volumes receiving doses over the prescription dose for AWBRT. All treatment plans were created on Oncentra TPS with two open tangential 6 MV photon beams from a Varian DHX-S linear accelerator with 120 MLC (Varian Medical System, Palo Alto, CA). Beam angles were chosen such that the posterior edges matched those of the corresponding TD treatment beams. Then, for each tangent, one sub-field was created with the MLC shaped to the 105% isodose and thereafter the relative weights of the open fields and the sub-fields were manually adjusted to achieve a uniform dose distribution within the PTV. Final dose calculation employed a beam modeling based on the collapsed cone superposition of points kernels [8]. The different treatment techniques have been applied to the patients’ dataset without any clinical application. This activity does not require an ethical approval according to our institution’s rules.

### Dose prescription and planning evaluation

A prescription dose of 50 Gy in 25 fractions to 50% of PTV was chosen. The PTV dose distribution was investigated evaluating the percentage volume of the PTV receiving 95% (V_95%_), more than 105% (V_105%_) and 107% (V_107%_) of the prescribed dose and PTV maximum dose (D_1%_).

A radiation conformity number (NC) defined as van’t Riet *et al.* formulation [9]

(1) $$\mathrm{NC}=\frac{TV_{\mathrm{RI}}}{\mathrm{TV}}\cdot \frac{TV_{\mathrm{RI}}}{V_{\mathrm{RI}}}$$

where TV is the PTV volume, TV_RI_ the target volume covered by the 95% isodose line and V_RI_ the volume encompassed by this isodose, was calculated to evaluate the target dose conformality. NC value ideally is equal to 1.

Moreover, to assess the target dose homogeneity, an homogeneity index HI, defined as [10]

(2) $$\mathrm{HI}=\frac{D_{95}}{D_{5}}$$

where D_5%_ and D_95%_ represent the dose to 5% and 95% of the PTV volume, was calculated. Low HI values indicate poor homogeneity.

Plan evaluation parameters for OARs were V_20Gy_, V_10Gy_, V_5Gy_ and mean lung dose (MLD) for ipsilateral lung; V_25Gy_ and mean heart dose (MHD) for heart; mean dose (D_mean_) and maximum dose (D_0.1cc_) for contralateral breast; maximum dose (D_0.1cc_) for controlateral lung. Excess irradiation (D_2cc_), defined as the percentage of the prescription dose delivered to a volume of 2 cc of the normal tissues external to the PTV, was analyzed. Finally, plans were compared with respect to the overall beam-on time. To define specific patient-class solutions to employ TD-3DCRT, correlations between dose constraints and patient specific parameters were tested. In particular, lung V_20Gy_ and MLD were correlated with Central Lung Distance (CLD), defined as the distance between the deep field edge to the thoracic wall in the central axis plane [11]; heart V_25Gy_ and MHD were correlated with the maximal heart distance (HD), measured as the maximum width of heart in the tangent fields [11]. Moreover, ipsilateral lung V_30Gy_ and V_40Gy_ were also investigated for TD-3DCRT-allocated patients.

### Statistical analysis

Statistically significant differences in dosimetric endpoints between TD-3DCRT and other two techniques were determined using the Wilcoxon signed-rank test for paired samples. Differences were considered significant for p < 0.05.

## Results

**Figure 1:**
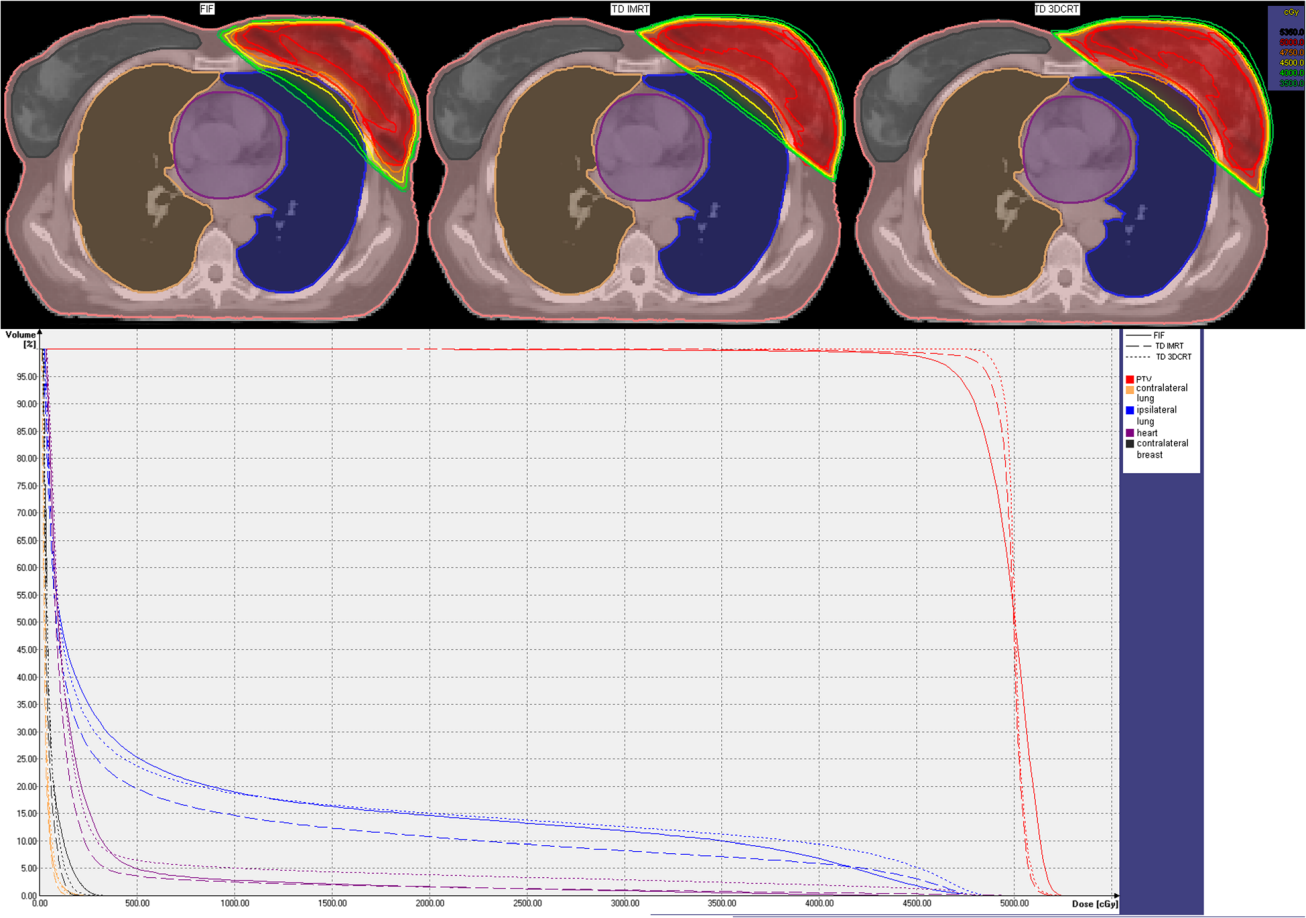
PTV and OARs cumulative DVHs for the 3 techniques.

Mean PTV volume was 731 cm^3^ (range 425–1643 cm^3^). PTV and OARs cumulative dose volume histograms (DVHs) and isodose distributions for a typical left-sided breast plan are illustrated in Figure 1. 

The mean values (± SD) of the PTV dosimetric endpoints of TD-3DCRT, TD-IMRT and FIF techniques are listed in Table 1. TD-3DCRT showed a whole breast coverage significantly better than TD-IMRT and FIF (p < 0.001): the V_95%_ was more than 99% in all plans. V_105%_ did not show significative difference among the techniques. However, V_107%_ and D_1%_ were significantly lower for the TD-3DCRT technique than FIF (p < 0.05 and p < 0.01, respectively), while no significative difference were registered between TD-3DCRT and TD-IMRT. 

**Table 1:** PTV dosimetric endpoints of TD-3DCRT, TD-IMRT and FIF techniques for AWBRT

**Endpoint**	**Technique**	**Value**	**p**
**V**_**95%**_**(%)**	**TD-3DCRT**	99.9 ± 0.1	
	**TD-IMRT**	98.9 ± 1.2	p < 0.001
	**FIF**	92.3 ± 2.1	p < 0.001
**V**_**105%**_**(%)**	**TD-3DCRT**	0.2 ± 0.3	
	**TD-IMRT**	0.3 ± 0.3	NS
	**FIF**	0.5 ± 0.5	NS
**V**_**107%**_**(%)**	**TD-3DCRT**	0.1 ± 0.2	
	**TD-IMRT**	0.1 ± 0.1	NS
	**FIF**	0.1 ± 0.5	p < 0.05
**D**_**1%**_(%)*	**TD-3DCRT**	3.2 ± 0.8	
	**TD-IMRT**	3.4 ± 0.8	NS
	**FIF**	4.6 ± 1.0	p < 0.01
**HI**	**TD-3DCRT**	0.965 ± 0.006	
	**TD-IMRT**	0.954 ± 0.011	p < 0.01
	**FIF**	0.906 ± 0.012	p < 0.001
**NC**	**TD-3DCRT**	0.618 ± 0.062	
	**TD-IMRT**	0.620 ± 0.067	NS
	**FIF**	0.651 ± 0.072	p < 0.05

Statistically significant differences between TD-3DCRT and the other techniques were considered significant for p < 0.05.

*as percentage of Rx.

Two-tailed *p* values from paired Wilcoxon test.

Data presented as mean ± standard devation.

The PTV homogeneity of TD-3DCRT plans was significantly better than TD-IMRT (p < 0.01) and FIF (p < 0.001); the PTV conformality was significantly less than FIF (p < 0.05), while no differences were found with respect to TD-IMRT.

The mean values (± SD) of the OARs dosimetric endpoints for each technique are listed in Table 2. Mean values of OARs dosimetric endpoints were significantly higher for TD-3DCRT than TD-IMRT. Compared to FIF, TD-3DCRT provided significantly higher vaues of ipsilateral lung mean volume receiving > 20 Gy (p < 0.05), mean heart volume receiving > 25 Gy (p < 0.01) and MHD (p < 0.01), while contralateral lung D_max_ and D_mean_ were significantly lower (p < 0.001). D_2cc_ was significantly higher for TD-3DCRT than TD-IMRT and FIF (p < 0.001). 

**Table 2:** OARs dosimetric endpoints of TD-3DCRT, TD-IMRT and FIF techniques for AWBRT

**Structure**	**Endpoint**	**Technique**	**Value**	**p**
**ISPILATERAL LUNG**	**V**_**5Gy**_**(%)**	**TD-3DCRT**	21.6 ± 5.3	
		**TD-IMRT**	18.7 ± 4.2	p < 0.01
		**FIF**	22.4 ± 5.2	NS
	**V**_**10Gy**_**(%)**	**TD-3DCRT**	16.8 ± 4.6	
		**TD-IMRT**	13.9 ± 3.3	p < 0.01
		**FIF**	15.8 ± 4.2	NS
	**V**_**20Gy**_**(%)**	**TD-3DCRT**	13.2 ± 4.1	
		**TD-IMRT**	9.9 ± 2.3	p < 0.001
		**FIF**	11.7 ± 3.6	p < 0.05
	**MLD (Gy)**	**TD-3DCRT**	6.9 ± 1.8	
		**TD-IMRT**	5.6 ± 1.1	p < 0.001
		**FIF**	6.3 ± 1.5	NS
	**V**_**30Gy**_(%)*	**TD-3DCRT**	6.5 ± 2.3	
		**TD-IMRT**	6.9 ± 1.5	NS
		**FIF**	7.3 ± 2.2	NS
	**V**_**40Gy**_(%)*	**TD-3DCRT**	5.3 ± 1.8	
		**TD-IMRT**	4.5 ± 1.2	NS
		**FIF**	4.2 ± 1.8	p < 0.05
**HEART**	**V**_**25Gy**_**(%)**	**TD-3DCRT**	5.5 ± 4.6	
		**TD-IMRT**	3.4 ± 3.1	p < 0.01
		**FIF**	3.4 ± 3.6	p < 0.01
	**MHD (Gy)**	**TD-3DCRT**	4.0 ± 2.2	
		**TD-IMRT**	3.0 ± 1.4	p < 0.05
		**FIF**	3.0 ± 1.6	p < 0.01
**CONTRALATERAL LUNG**	**D**_**0.1cc**_**(Gy)**	**TD-3DCRT**	1.8 ± 0.6	
		**TD-IMRT**	1.5 ± 0.5	p < 0.01
		**FIF**	2.3 ± 0.9	p < 0.001
**CONTRALATERAL BREAST**	**D**_**0.1cc**_**(Gy)**	**TD-3DCRT**	3.4 ± 1.9	
		**TD-IMRT**	2.3 ± 1.3	p < 0.05
		**FIF**	4.3 ± 3.0	NS
	**D**_**mean**_**(Gy)**	**TD-3DCRT**	0.43 ± 0.09	
		**TD-IMRT**	0.38 ± 0.07	p < 0.05
		**FIF**	0.52 ± 0.12	p < 0.001
**Normal tissues external to PTV**	**D**_**2cc**_**(%)**^**^**^	**TD-3DCRT**	8.3 ± 2.3	
		**TD-IMRT**	5.0 ± 1.4	p < 0.001
		**FIF**	4.4 ± 1.3	p < 0.001

Statistically significant differences between TD-3DCRT and the other techniques were considered significant for p < 0.05.

*Volumes of ipsilateral lung receiving 30 Gy and 40 Gy were calculated for TD-3DCRT plan class patients only.

^as percentage of Rx.

Two-tailed *p* values from paired Wilcoxon test.

Data presented as mean ± standard devation.

**Figure 2:**
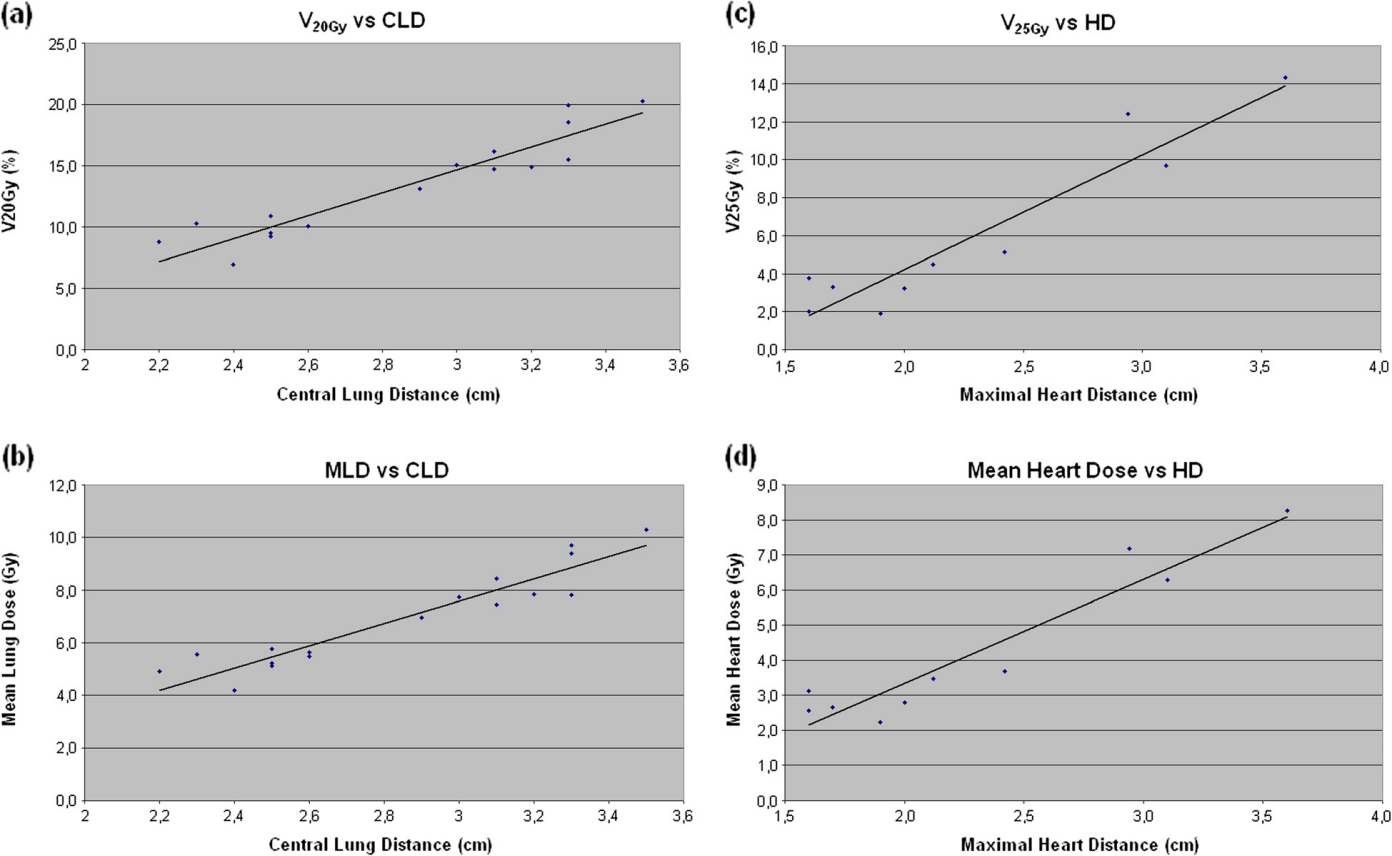
Regression plots of: (a) lung V_**20Gy **_vs. CLD; (b) MLD vs. CLD; (c) heart V_**25Gy **_vs. HD; (d) MHD vs. HD.

The correlation between CLD and ipsilateral lung dose was statistically significant for both V_20Gy_ and MLD, with Spearman’s ρ = 0.909 (p < 0.0001) and 0.925 (p < 0.0001), respectively (Figure 2). Similarly, HD correlated with heart V_25Gy_ and MHD, with Spearman’s ρ = 0.827 (p < 0.004) and 0.851 (p < 0.002) (Figure 2). 

The setting of the compensation factor did not significantly affect the dose metrics both for PTV and OARs. Deselecting the normal tissue homogeneity option, increased the volume encompassed by the 95% isodose line and therefore significantly reduced the radiation conformity number from 0.622 to 0.541 (p < 0.05), as increased the D_2cc_ of approximately 4%.

The mean beam-on time for TD-3DCRT was 295 s (range 219–408 s), significantly shorter than TD-IMRT, with a mean time of 378 s (range 267–475 s, p < 0.001). The use of the low compensation factor produced a mean reduction of beam-on time of approximately 15%.

## Discussion

While TD-IMRT has already been studied, TD-3DCRT still deserves investigation. In the preliminary study of Reynders et al. [5], TD-IMRT showed excellent PTV coverage and OARs sparing compared to conventional 3DCRT and HT. However, a dose inhomogeneity within the PTV (hotspots surpassing 115% of the prescribed dose) was reported, potentially calling for multiple fields with a consequent increase in treatment time. Schubert et al. [6] compared TD-IMRT to conventional 3DCRT: both modalities provided adequate PTV coverage, but TD-IMRT resulted in a significant reduction of high doses to normal tissue.

TD-3DCRT allows intensity modulation with lower resources request in terms of planning and treatment time compared to TD-IMRT. Specifically, TD-3DCRT employs a preset optimization algorithm, only requiring the specification of beam angles, target volumes and dose prescriptions. Consequently, planning and calculation time are consistently lower and comparable to those of conventional 3DCRT. Conversely, beam-on time resulted in a 22% average decrease, which can be further reduced to less than 4 min using the compensation factor low. These considerations make TD-3DCRT very attractive, mainly for radiotherapy departments without conventional Linacs and only equipped with helical tomotherapy units. To evaluate whether this technique might represent a viable solution, we compared 17 TD-3DCRT plans to TD-IMRT and conventional FIF 3DCRT, focusing on PTV and OARs dosimetric endpoints.

**Figure 3:**
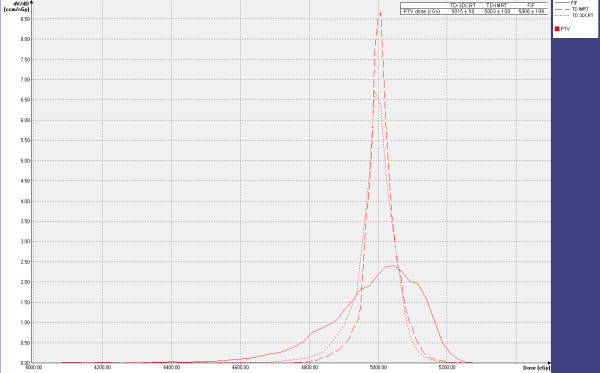
Differential PTV DVHs for the techniques.

The results show that TD-3DCRT provided slightly better PTV coverage and dose homogeneity, as underlined in Figure 3, reporting PTV differential DVHs of a sample patient. This can be explained taking into account that in 3DCRT mode, dosimetric constraints are limited to prescription dose in the target as a dose-volume-histogram (DVH) point. Considering PTV average dose and corresponding SDs, TD-3DCRT DVH provided the most reduced width and skew, whilst FIF DVH is skewed towards high dose volumes. Conversely, the symmetry of the PTV differential DVH shows the different PTV homogeneity level achieved by the 3 techniques. Dose to 1% of the PTV was found substantially equal for TD-3DCRT and TD-IMRT, pointing out similar efficacy in limiting maximal dose within the prescription volume. 

The dosimetric concern with TD-3DCRT for AWBRT treatments is mainly represented by the radiation dose delivered to the OARs: as shown by our results, both lungs, heart (for left-sided breast cancer patients) and contralateral breast doses are significantly higher than those obtained with TD-IMRT planning. This is particularly evident for ipsilateral lung, if MLD or V_20Gy_ are considered and for heart if MHD or V_25Gy_ are taken into account (see Table 2 for details). As a consequence, the use of TD-3DCRT in the context of AWBRT might not necessarily be generalized and applied to every typology of patients.

Hence, the definition of specific patient-class solutions, obtained correlating the OARs dose constraints with patient specific anatomic parameters such as the CLD and the HD, might be extremely helpful in creating a subset of patients better suitable for TD-3DCRT, in which the intrinsic spatial relationship between target volume and normal tissues might be able to compensate for the eventual dosimetric slight disadvantage, with no consequent detrimental effects.

Radiation-induced lung injury (RILI) variously occurs (1%-50%) after AWBRT according to different series (prospective or retrospective) and to the heterogeneity of the scoring systems and clinical endpoints employed [12]. If clinically significant radiation pneumonitis (RP) is considered, it may be observed in 1–5% of cases, whereas subclinical RILI (pulmonary function tests worsening/radiological changes) is more frequent [13]. MLD and V_20Gy_ are reliable dosimetric predictors of RP [13]. Dosimetric threshold on this parameters are used in the planning scenario to decrease toxicity risk. [12]. AWBRT is frequently delivered to prevent from local recurrence good prognosis women; hence it is crucial to adopt safe dosimetric constraints. In our study, we obtained ipsilateral lung V_20Gy_ = 13.2% (V_20Gymax_ = 20.3%) and MLD = 6.9 Gy (MLD_max_ = 10.3 Gy) for TD-3DCRT, significantly higher than for FIF and TD-IMRT. In the different series correlating RP probability and dosimetric predictors, it is rather uncommon to observe an increase in RILI probability for V_20Gy_ < 20% and MLD < 10 Gy [12]. However, Tsujino et al. [14], evaluating 71 lung cancer patients treated with concurrent chemoradiation, reported 2 cases of Grade 1 RP (according to NCI-CTC v 2.0) for V_20Gy_ = 15%. The NKI and Duke University series [15,16], described few cases of RP for MLD < 10 Gy (grade 2 according to SWOG and all grade according to Duke University criteria, respectively). In details, the Duke University study reported a 10% occurrence rate of RP for MLD < 10 Gy, while the NKI study decribed 1 case of grade 2 RP in the breast/lymphoma cohort related to a biological MLD (NTD_mean_) of 8–12 Gy.

Hence, we chose 2 strict lung dosimetric parameters (V_20Gy_ < 15% and MLD < 8 Gy) to allocate patients to TD-3DCRT or TD-IMRT. CLD predicts lung volume involved within the treatment field during AWBRT. A standard tangential approach covers a 2–3 cm CLD measured on a simulation film. Das et al. estimated that 3–3.5 cm CLD represents a lung volume of 15–26% [17].

The risk of symptomatic RP calculated on CLD datasets is < 2% for a CLD range of 1–3 cm and up to 10% for CLD > 4 cm [18]. In our study we correlated V_20Gy_, MLD and CLD within a linear fit (Figure 2). A dosimetric threshold on V_20Gy_ = 15% and MLD = 8 Gy corresponds to a CLD cut-off of approximately 3 cm. This CLD value might be a useful clinical tool to predict the opportunity to employ TD-3DCRT (for CLD < 3 cm) or TD-IMRT (for CLD ≥ 3 cm) according to anatomical characteristics to minimize RILI. To further test the safety of TD-3DCRT in terms of lung dose, we selected the 9 patients allocated to TD-3DCRT according to CLD value and analyzed ipsilateral lung V_30Gy_ and V_40Gy_. No differences were found between TD-3DCRT and TD-IMRT, confirming the equivalence also at medium-range lung doses (Table 2).

Radiation-induced myocardial damage involves several components (pericardium, valvular and conduction systems, myocardium and coronary arteries).

The dose–response relationship for cardiac risk has been extensively studied, but several uncertainties still remain. The relative seriality model and long-term cardiac mortality clinical data from Scandinavian trials, lead to a threshold dose of 20 Gy for ischaemic disease death [19]. Adjunctively, for conventionally fractionated partial heart irradiation, the same conservative NTCP model-based estimate predicts that V_25Gy_ < 10% corresponds to a cardiac death probability < 1% at 15 years [19]. Conversely, a study by Carr et al. [20] demonstrated a coronary artery disease relative risk increase in peptic ulcer patients whose cardiac apex exceeded 12 Gy (estimated MHD > 2.6 Gy).

**Figure 4:**
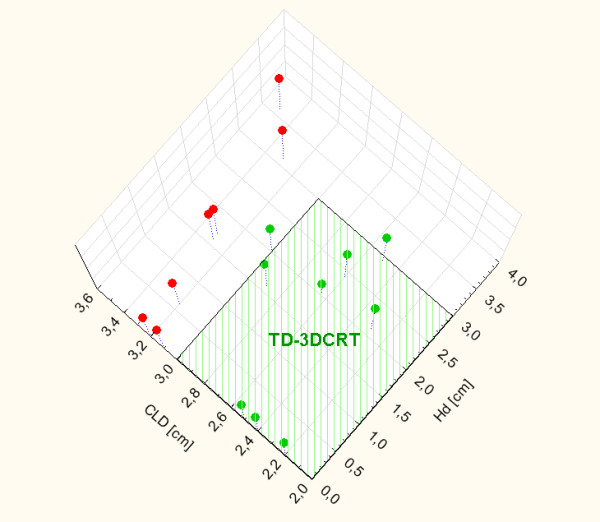
Allocation pattern of the 17 patients according to CLD and HD.

This finding is in line with atomic bomb survivors data where a cardiac mortality increase was documented for 0–4 Sv whole body uniform doses (roughly corresponding to 0–4 Gy, since gamma rays irradiation has a negligible neutron component), with a threshold value of approximately 0.75 Sv [21]. MHD strongly correlates to normal tissue complication probability (NTCP) in terms of cardiac-morality, for left-sided AWBRT [22]. The present study showed a heart V_25Gy_ range of 1.9–14.3% for TD-3DCRT, higher than for others techniques. Figure 2 plots V_25Gy_ as a function of HD for TD-3DCRT. A linear correlation between V_25Gy_ and HD was found with a possible 3 cm threshold for HD to properly allocate patients to TD-3DCRT considering cardiac toxicity risk (TD-IMRT for HD ≥ 3 cm). Conversely, MHD did not seem to be a clinically useful screening method for patient allocation. In this sense, a safe dosimetric threshold (MHD < 2.6 Gy) would lead to a HD of roughly 1.5 cm forcing patient planning with TD-IMRT. A permissive dosimetric threshold (MHD < 20 Gy) would allow for a TD-3DCRT planning for the whole cohort. The allocation pattern for all patients according to CLD (>3 cm) and HD (>3 cm) is shown in Figure 4. Two patients (12%) presented both CLD and HD > 3 cm, while 5 patients (29%) showed a CLD > 3 cm. Consequently, 10 patients (59%) were potentially allocable to TD-3DCRT. However, 2 issues should be considered to definitively allocate patients: normal tissues dose hotspots external to PTV and contralateral breast dose. TD-3DCRT showed a significantly higher maximum dose outside the PTV (mean value = 8.3% of the prescription dose). These hotspots do not represent a consistent issue, since they involve a very limited volume outside PTV and they do not exceed 10% of the prescribed dose, a threshold for acute skin radiation-induced toxicity during AWBRT [23]. To further reduce dose hotspots in beam entrance and exit regions, one can use multiple beams at different angles or contour ad-hoc planning helping structures, but at the cost of overall planning time increase or eventual higher dose to OARs. Hence we decided to keep planning procedure agile. The dose homogeneity option was always selected, since it minimizes hotspots within normal tissues and its deselection reduced the radiation conformity number without benefit. 

Regarding the contralateral breast, TD-3DCRT provided mean D_0.1cc_ values < 5 Gy (Table 2); however it provided higher doses than TD-IMRT, with a contralateral breast D_max_ range of 0.9–6.7 Gy, similar to conventional 3DCRT. Boice et al. investigated secondary contralateral breast cancer, reporting a relative risk of 1.19 of developing second breast cancer after previous AWBRT (estimated average radiation dose = 2.82 Gy, Dmax 7.1 Gy) [24]. Thus, a dose range between 4–5 Gy to the contralateral breast might be considered sufficiently safe in terms of radiogenic second malignancies and consequently in the present study we focused on a cut-off dose of 5 Gy to definitely allocate patients to TD-3DCRT (contralateral breast dose < 5 Gy) or to replan with TD-IMRT (contralateral breast dose ≥ 5 Gy). Consequently, in our study, the number of patients allocated to TD-3DCRT was finally reduced to 7 (41%).

## Conclusions

TD-3DCRT and TD-IMRT are two feasible, reliable and dosimetrically acceptable treatment approach to deliver AWBRT, with optimal PTV coverage and adequate OARs sparing. The lower request of resources in terms of planning, calculation and beam-on time of TD-3DCRT makes this solution very attractive. However some concerns should be raised regarding eventual undue dose delivered to OARs (lungs and heart in particular) if TD-3DCRT is applied to a general patient population. A correct patients clusterization according to simple quantitative anatomic measures (such as HD and CLD), would help to correctly allocate patients to the appropriate treatment strategy in terms of target coverage, but also of normal tissue sparing.

## Competing interests

The authors declare that they have no competing interests.

## Authors’ contributions

All authors contributed to drafting the manuscript and all authors reviewed and approved the final manuscript.
